# Supplementary material for: Structure–activity studies of Streptococcus pyogenes enzyme SpyCEP reveal high affinity for CXCL8 in the SpyCEP C-terminal
Source: Sci Rep. 2023 Nov 3;13:19052. doi: 10.1038/s41598-023-46036-9 (PMC10624844; doi:10.1038/s41598-023-46036-9)
Supplement: Supplementary file 1 — Supplementary Figures. [file 41598_2023_46036_MOESM1_ESM.docx]

**Supplementary appendix**

**Structure-activity studies of *Streptococcus pyogenes* enzyme SpyCEP reveal high affinity for CXCL8 in the SpyCEP C-terminal**

**Max Pearson^1,2^, Carl Haslam^3^, Andrew Fosberry^3^, Emma J Jones^3^, Mark Reglinski^1^, Lucy Reeves^1^, Robert J. Edwards^4^, Richard Ashley Lawrenson^1^, Jonathan C Brown^1^, Danuta Mossakowska^3,5^, James Edward Pease*^6^, Shiranee Sriskandan*^1, 2^**

^1^Department of Infectious Disease, Imperial College London, London W12 0NN, UK

**^2^**Centre for Bacterial Resistance Biology, Imperial College London, London, SW7 2AZ, UK

^3^GlaxoSmithKline R&D, Gunnels Wood Road, Stevenage, Hertfordshire, SG1 2NY, UK

^4^Department of Medicine, Imperial College London, W12 0NN, UK

^5^Malopolska Centre of Biotechnology, Jagiellonian University, 30-387 Kraków, Poland

^6^National Heart and Lung Institute, Imperial College London, London, SW7 2AZ, UK

*Corresponding authors

#
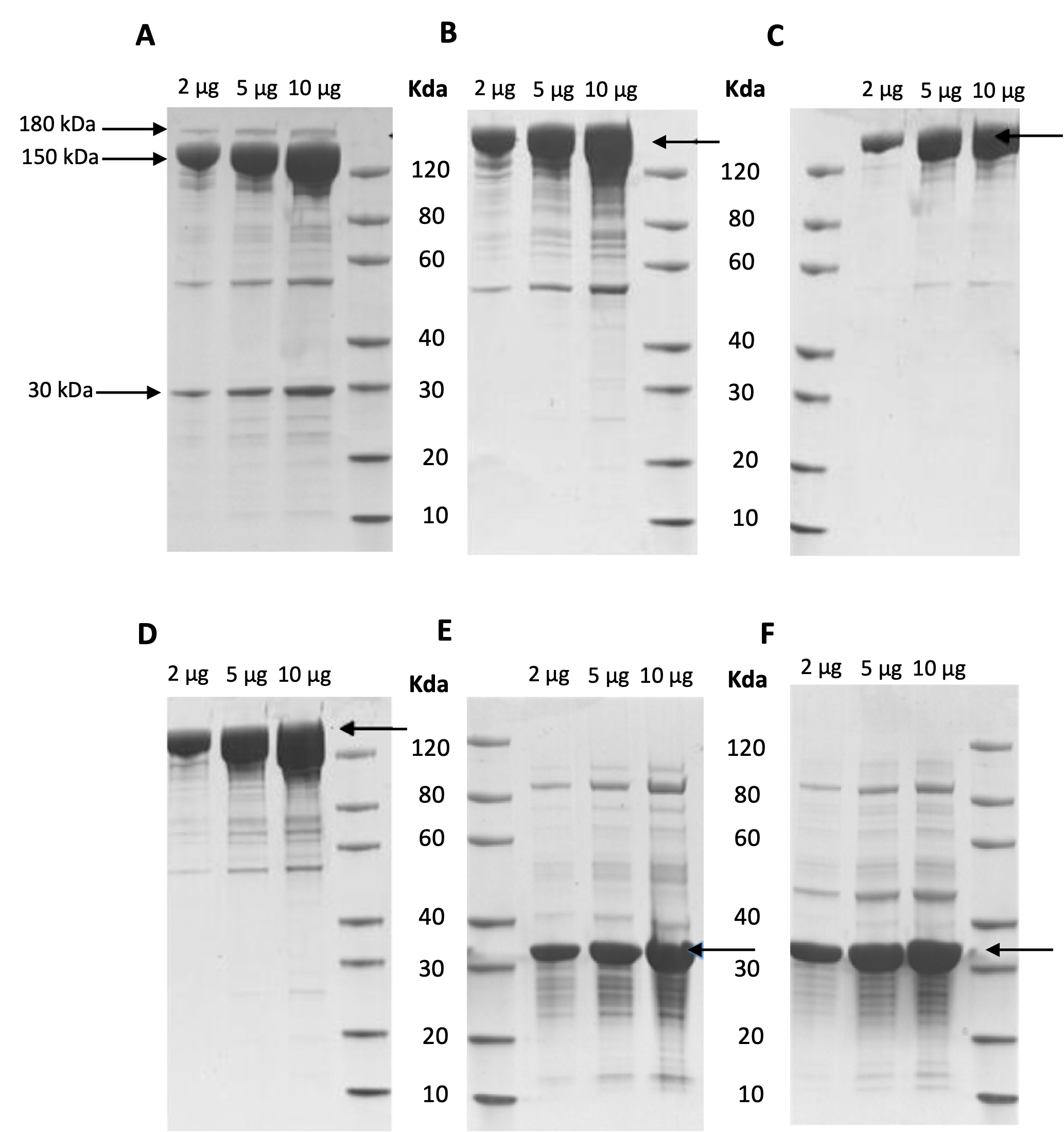
Supplementary figures

**Figure S1. SDS-PAGE analysis of recombinant SpyCEP constructs cloned and expressed in *Escherichia coli.***

SDS-PAGE gels of 2, 5 or 10 μg of *E. coli* expressed recombinant SpyCEP constructs **A.** Full-length, SpyCEP^34-1613^-6His. **B.** Full-length double mutant, SpyCEP^34-1613 D151A, S617A^-6His. **C.** C-terminal fragment, SpyCEP^245-1613^-6His. **D.** C-terminal mutant fragment, SpyCEP^245-1613 S617A^-6His. **E.** N-terminal fragment, FLAG-TEV-SpyCEP^34-244^. **F.** N-terminal mutant fragment, FLAG-TEV-SpyCEP^34-244 D151A^. Arrows highlight banding of multiple size SpyCEP constructs at 180 kDa, 150 kDa and 30 kDa. Additional banding are non-specific *E. coli* products.


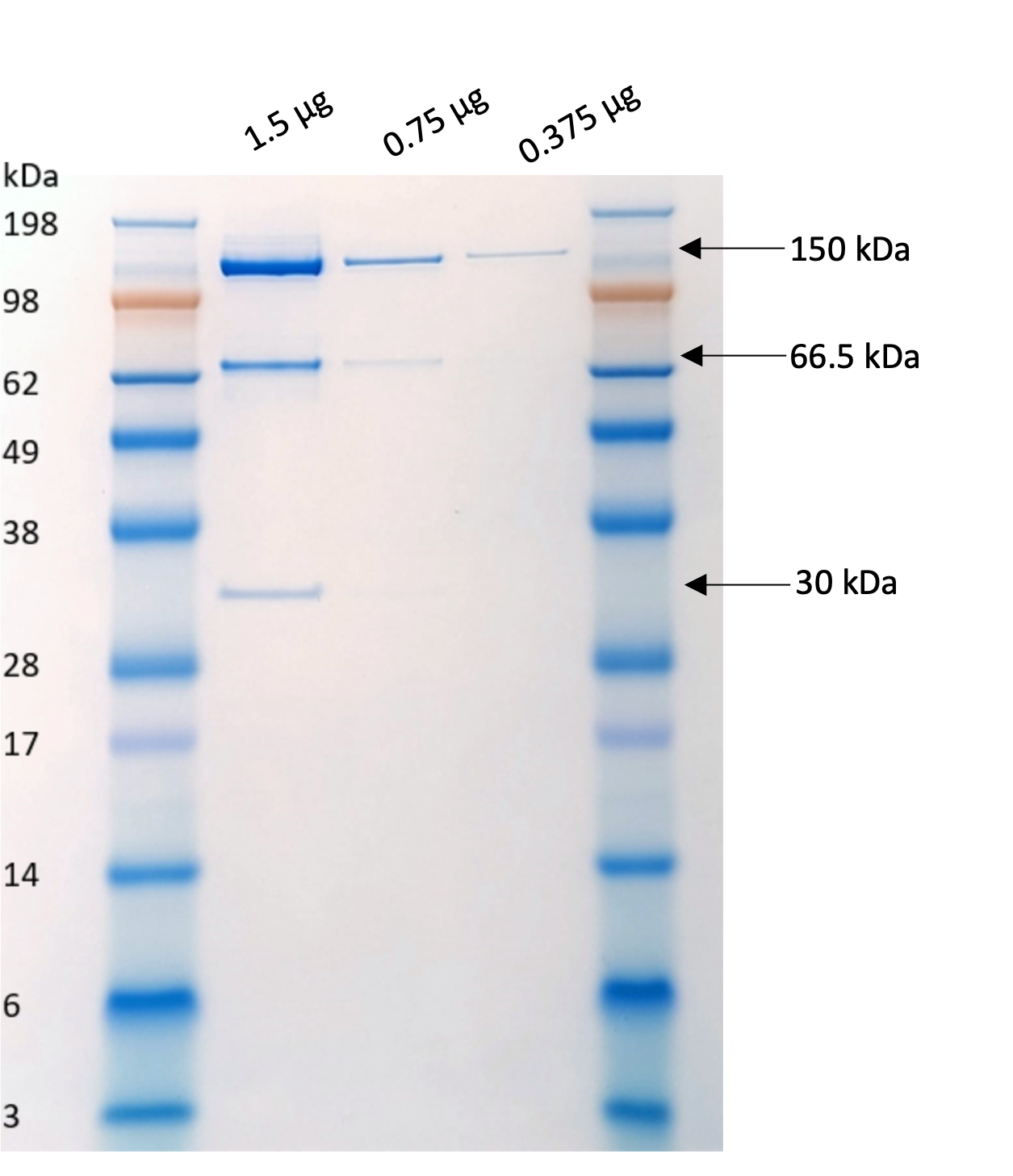


**S2. SDS-PAGE analysis of His-tagged recombinant *S. pyogenes* SpyCEP (s.pSpyCEP)**

SDS-PAGE gel of 1.5, 0.75 and 0.375 μg of s.pSpyCEP expressed in and purified from *S. pyogenes.* Arrows highlight banding of multiple size SpyCEP constructs at 150 kDa and 30 kDa. Banding at 66.5 kDa represents BSA carrier protein.


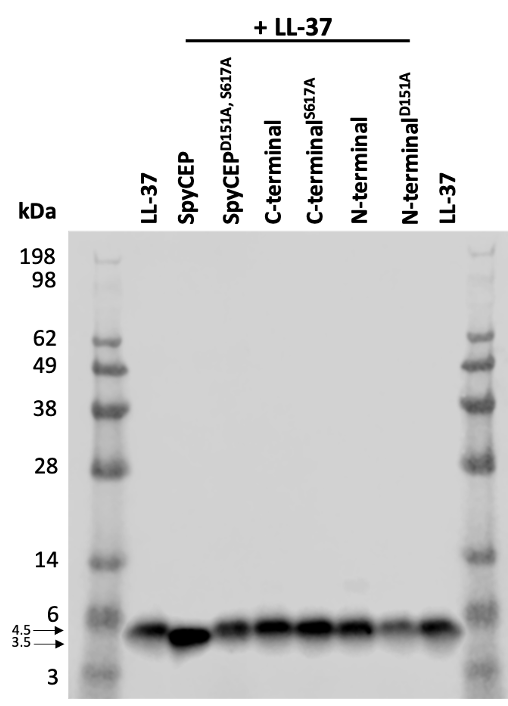


**Figure S3.** **Western blot of SpyCEP specific LL3-7 cleavage.** Immunoblot of 111.1 pmol human LL-37 incubated for 16 hours at 37 ^o^C either alone (lane 1 and 8) or with a panel of SpyCEP constructs at a 10:1 molar ratio in favour of LL-37. 4.5 kDa full-length LL-37 band and 3.5 kDa cleaved LL-37 band are indicated by arrows and were detected with 2 μg/ml sheep IgG polyclonal LL-37 antibody and rabbit anti-sheep IgG antibody (1:40,000). Figure is representative of two experiments.


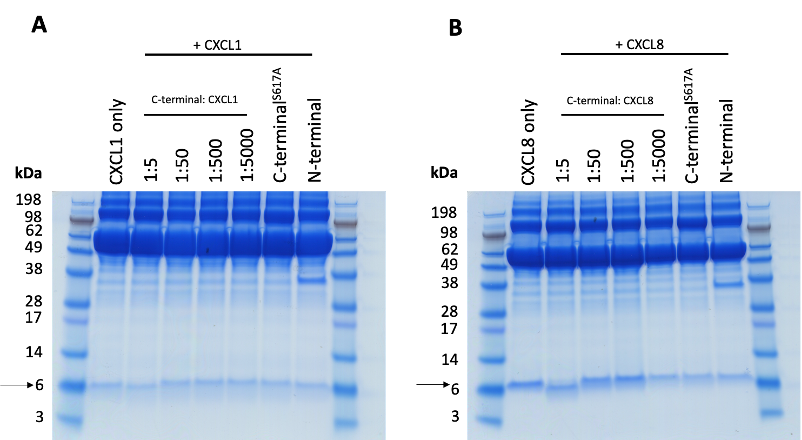


**Figure S****4. Comparative cleavage of CXCL1 and CXCL8 by the C-terminal of SpyCEP.** SDS-PAGE analysis showing cleavage of A. 50 pmol human CXCL1 (all lanes) or B. 50 pmol human CXCL8 (all lanes) by C-terminal SpyCEP at a 1:5 – 1:5000 molar ratio (SpyCEP: Chemokine). C-terminal^S617A^ and N-terminal controls were both assayed at the highest 1:5 molar ratio. CXCL1 and CXCL8 bands are indicated by arrows; cleavage is indicated by shift in molecular weight. Note that high molecular weight bands present in all lanes are from carrier protein present in SpyCEP: chemokine reaction mixtures.


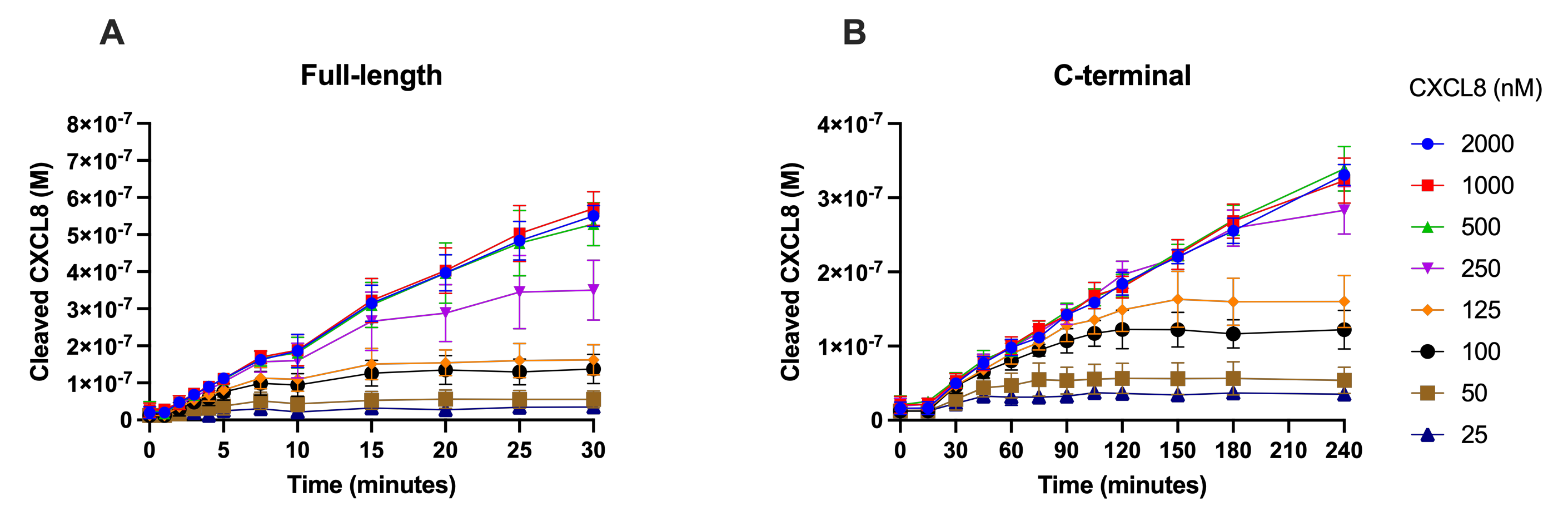


**Figure S5. CXCL8 cleavage by active recombinant SpyCEP constructs using mass spectrometry**. **A.** Production of the 13 amino acid species cleaved from CXCL8 by 250 pM of full-length SpyCEP over 30 minutes at room temperature, following incubation with 6.25 nM – 2000 nM CXCL8, 1: 250 – 8000 molar ratio (SpyCEP: CXCL8). **B**. Production of the 13 amino acid species by 40 nM SpyCEP C-terminal fragment over a 4 hour room temperature incubation, following incubation with 6.25 nM – 2000 nM CXCL8, 1: 0.15 – 50 molar ratio (SpyCEP: CXCL8). N=5 experimental replicates per data point, error bars represent SD. Figure is representative of two experiments.


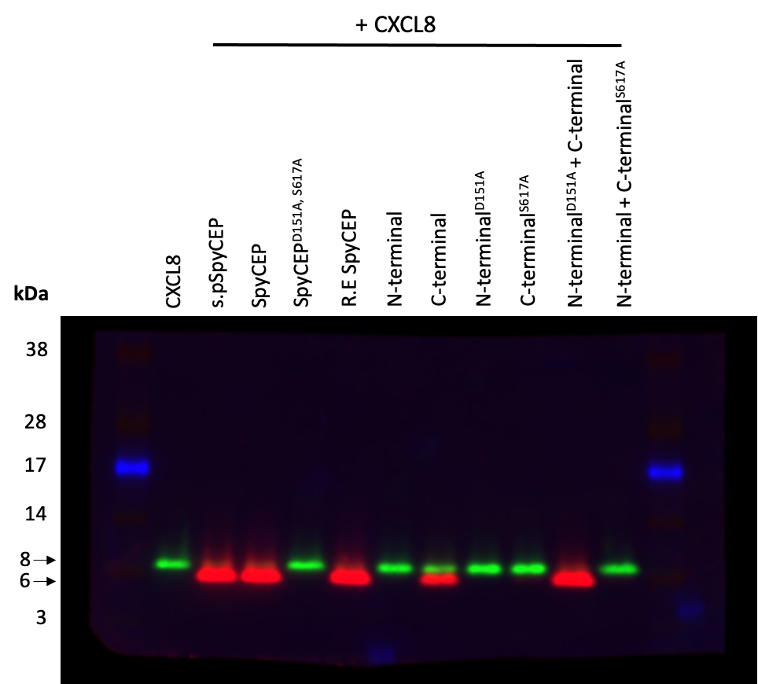


**Figure S6. Cleavage activity of recombinant SpyCEP constructs assayed by immunoblot.** Two colour immunoblot showing cleavage of 18.75 pmol CXCL8 when incubated for 2 hours at 37 ^o^C either alone (2^nd^ lane) or with a panel of SpyCEP constructs at a 1:50 molar ratio (SpyCEP: CXCL8). Green bands represent intact CXCL8 (anti-CXCL8 antibody); red bands represent cleaved CXCL8 (anti- ENWVQ). A 17 kDa molecular weight marker is shown in blue, 8 kDa and 6 kDa molecular weights are highlighted by arrows. Figure is representative of 2 independent immunoblots.
